# High Serum Phosphate Level as a Risk Factor to Determine Renal Prognosis in Autosomal Dominant Polycystic Kidney Disease: A Retrospective Study

**DOI:** 10.3390/medicines7030013

**Published:** 2020-03-12

**Authors:** Masayo Sato, Hiroshi Kataoka, Yusuke Ushio, Shun Manabe, Saki Watanabe, Taro Akihisa, Shiho Makabe, Rie Yoshida, Naomi Iwasa, Michihiro Mitobe, Norio Hanafusa, Ken Tsuchiya, Kosaku Nitta, Toshio Mochizuki

**Affiliations:** 1 Department of Nephrology, Tokyo Women’s Medical University, Tokyo 162-8666, Japan; 2 Clinical Research Division for Polycystic Kidney Disease, Department of Nephrology, Tokyo Women’s Medical University, Tokyo 162-8666, Japan; 3 Department of Blood Purification, Kidney Center, Tokyo Women’s Medical University, Tokyo 162-8666, Japan

**Keywords:** phosphate, polycystic kidney disease, *fibroblast growth factor 23*, *klotho*, renal prognosis

## Abstract

**Background:** Serum phosphate levels, which are associated with the progression of renal dysfunction in chronic kidney disease, in patients with autosomal dominant polycystic kidney disease (ADPKD) are lower than those in patients with other kidney diseases. However, their role in ADPKD remains unclear. This study aimed to determine whether serum phosphate levels could have an association with renal prognoses among patients with ADPKD. **Methods:** In total, 55 patients with *PKD1* or *PKD2* mutations but not undergoing dialysis were evaluated. Data regarding serum phosphate levels were collected, and Cox regression analyses were used to calculate hazard ratios (HRs) with renal replacement therapy as the endpoint. **Results:** The median (quartile 1; quartile 3) serum phosphate concentration was 3.4 (3.1; 3.9) mg/dL, and the estimated glomerular filtration rate (eGFR) was 39.5 (17.6; 65.7) mL/min/1.73 m^2^. The multivariate analysis that included age, *PKD1* mutation, eGFR, urinary protein excretion, hyperuricemia, and serum phosphate determined that eGFR (HR, 0.82; 95% confidence interval (CI), 0.74–0.90; *p* < 0.0001) and serum phosphate (HR, 6.78; 95% CI, 1.94–34.02; *p* = 0.0021) were independently associated with renal replacement therapy. **Conclusions:** We found that serum phosphate levels were significantly associated with poor renal prognoses in patients with ADPKD.

## 1. Introduction

Autosomal dominant polycystic kidney disease (ADPKD), one of the most common hereditary kidney diseases, causes a gradual growth of cysts in the kidneys, which leads to renal failure [1]. The two causative genes are *PKD1* and *PKD2,* and end-stage renal disease (ESRD) tends to occur around 53 years of age in patients with *PKD1* and 68 years of age in patients with *PKD2*, respectively [2,3]. Recently, we reported that renal prognosis differed according to mutation types: it was poor for those with *PKD1* splicing, *PKD1* frameshift, and *PKD2* splicing mutations, and it was relatively favorable for those with nonsense mutations among patients with *PKD1* truncating mutations [4]. Additional factors affecting the progression of renal dysfunction in patients with ADPKD include male sex, diagnosis and gross hematuria before 30 years of age, development of hypertension before 35 years of age, anemia, higher levels of urinary sodium excretion, and a higher 24-h urine osmolality at baseline [2,3,5,6]. Especially, we recently reported that anemia might be a factor for poor renal prognosis in ADPKD in association with a sex difference [6].

Predicting renal outcomes from the ADPKD score, which considers genetic and environmental factors but does not account for the serum phosphate levels, has been proposed as a means to predict renal prognoses and to help create individual monitoring and treatment plans [3]. When considering serum phosphate levels, increases are first observed at chronic kidney disease (CKD) stage 4, and high levels are associated with poor renal prognoses [7]. Although the serum phosphate levels in ADPKD are lower than those in other kidney diseases [8], little is known about the relationship between the serum phosphate level and renal prognosis in people with ADPKD.

Therefore, this study aimed to evaluate the genetic and environmental factors related to renal prognoses in a cohort of individuals genetically diagnosed with ADPKD who underwent measurements of their serum phosphate levels during their initial examinations to determine whether there is an association between serum phosphate levels and renal prognoses.

## 2. Materials and Methods

### 2.1. Study Design 

All procedures performed in this study were approved by the research ethics committee of the Tokyo Women’s Medical University (No. 196B; Date of approval: 4 February 2015) in accordance with the 1964 Helsinki Declaration and its later amendments, or with comparable ethical standards, and written informed consent was obtained from all individual participants included in the study.

**Figure 1:**
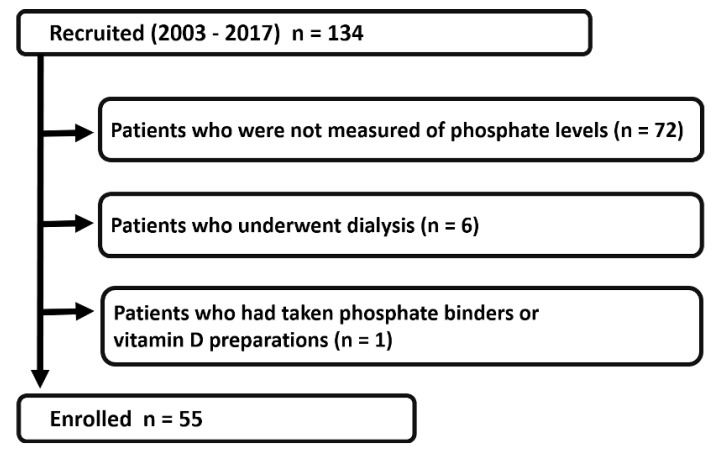
Flow chart of patient selection. From 134 patients screened initially, 72 who missed data regarding phosphate levels, 6 who underwent dialysis, and 1 who had taken phosphate binders or vitamin D preparations were excluded; the remaining 55 patients were enrolled in this study.

We recruited 134 patients with ADPKD who visited the Tokyo Women’s Medical University Hospital, Japan, between November 2010 and June 2016. ADPKD was diagnosed using previously described criteria [9], and genetic analyses of all patients were available. Among these patients, those missing data regarding phosphate levels (n = 72), those who underwent dialysis (n = 6), and those who had taken phosphate binders or vitamin D preparations (n = 1) were excluded from the study. In total, kidney survival was retrospectively examined in 55 individuals with ADPKD and identified according to *PKD1* or *PKD2* mutation (Figure 1).

### 2.2. Covariate Assessments and Definitions of the Comorbidities

Clinical data from the initial examinations were used. The serum phosphate levels were obtained within 3 months of the initial examination. Hypertension was defined as systolic BP ≥ 140 mmHg, diastolic BP ≥ 90 mmHg, or taking an antihypertensive agent. Hyperuricemia was defined as a serum uric acid level ≥ 7 mg/dL or taking an antihyperuricemic agent. Dyslipidemia was defined as serum triglyceride (TG) level ≥ 150 mg/dL, serum HDL-C level ≤ 40 mg/dL, serum LDL-C level ≥ 140 mg/dL, or taking an antidyslipidemic agent. Diabetes was defined as HbA1c (standardized by the National Glycohemoglobin Standardization Program) ≥ 6.5% or taking an oral antidiabetic agent or insulin therapy.

### 2.3. Study End Point

The patients were examined retrospectively using renal replacement therapy (RRT) as the endpoint.

### 2.4. Statistical Analyses

Continuous variables were reported as medians with the first (Q1) and third (Q3) quartiles. Categorical variables were reported as numbers and percentages. The Mann–Whitney U or chi-square tests were used to compare the men and women. Univariate and multivariate Cox regression analyses based on RRT were used to calculate the hazard ratios (HRs) and 95% confidence intervals (CIs). Variables with *p*-values < 0.1 in the univariate model were included in the multivariate analyses. A value of *p* < 0.05 was considered statistically significant. JMP Pro software, version 14.0.0 (SAS Institute Inc., Cary, NC, USA), was used for all the statistical analyses.

## 3. Results

### 3.1. Patient Characteristics

Table 1 shows patient characteristics, clinical findings, and examination findings. The *PKD1* mutation was present in 40 patients (72.7%). The median (Q1; Q3) age was 49 (40; 59) years, and median (Q1; Q3) serum phosphate level was 3.4 (3.1; 3.9) mg/dL. Three patients (5.5%)—comprising one man and two women—had serum phosphate levels ≥ 4.5 mg/dL. The median (Q1; Q3) estimated glomerular filtration rate (eGFR) was 39.5 (17.6; 65.7) mL/min/1.73 m^2^. The CKD stages were stage 1 or 2 in 14 patients (25.5%), stage 3 in 20 patients (36.4%), and stage 4 or 5 in 21 patients (38.2%). The median (Q1; Q3) body mass index (BMI) was 22.1 (20.5; 23.4) kg/m^2^, and the serum albumin level was 4.2 (4.1; 4.5) g/dL. Thirty-nine patients (70.9%) had hypertension, while two (3.6%) had diabetes. There were significant differences between men and women in terms of BMI; eGFR; and serum creatinine, uric acid, triglyceride, and HDL-C levels; use of drugs, including antihypertensive agents, angiotensin receptor blockers, angiotensin-converting enzyme inhibitors, and antihyperuricemic agents; and the presence of hypertension, hyperuricemia, and hypertriglyceridemia.

### 3.2. Prognostic Indicators in Patients with ADPKD 

We performed univariate and multivariate Cox regression analyses to detect any associations between the baseline clinical findings and RRT during the follow-up period. At the follow-up examination in June 2018, 21 patients (38.1%) reached the endpoint. The univariate analyses showed that age (HR, 1.04; 95% CI, 1.01–1.08; *p* = 0.02); *PKD1* mutation (HR, 3.61; 95% CI, 1.02-22.96, *p* < 0.05); eGFR (1 mL/min/1.73m^2^ increase) (HR, 0.83; 95% CI, 0.76-0.89; *p* < 0.001); urinary protein excretion (HR, 1.69; 95% CI, 1.17–2.39; *p* = 0.01); hyperuricemia (HR, 3.09; 95% CI, 1.23–8.75; *p* = 0.02); and serum phosphate level (HR, 4.61; 95% CI, 2.14–10.27; *p* < 0.001) were significant risk factors associated with RRT. 

The multivariate analysis that included age, eGFR, *PKD1* mutation, urinary protein excretion, hyperuricemia, and serum phosphate showed that eGFR (HR, 0.82; 95% CI, 0.74–0.90; *p* < 0.001) and serum phosphate (HR, 6.78; 95% CI, 1.94–34.02; *p* = 0.002) were independently associated with RRT (Table 2).

## 4. Discussion

This study examined the genetic and environmental factors affecting renal prognoses in patients with ADPKD, limited to patients who had undergone genetic analyses. *PKD1* mutation is thought to be associated with a poor renal prognosis [2]; however, differences in progression are often present within families [10]. While ADPKD is a hereditary disease, it is autosomal dominant; therefore, its onset is subject to the ‘two-hit’ theory, hypomorphic mutations, and other acquired factors [1]. Hence, a variety of environmental factors may be involved in the progression of ADPKD. We focused on the serum phosphate levels in this ADPKD cohort.

The present study showed that elevated serum phosphate levels had an association with RRT among patients with ADPKD. When literature search was conducted by using keywords—renal prognosis, autosomal dominant polycystic kidney disease, and phosphate through the PubMed in March 2020, only two documents were selected. One was a case report of staghorn calculus, and the other was a review of vasopressin antagonists. To our knowledge, this is the first report to describe the association between serum phosphate and renal prognosis in patients with ADPKD. 

Although little is known about the relationship between the serum phosphate level and renal prognosis in people with ADPKD, several studies regarding CKD have shown that serum phosphate levels are related to the renal prognoses in patients with CKD [11,12,13]. A study of 448 non-dialysis patients with CKD stages 4 to 5 indicated that higher levels of serum phosphate were associated with a more rapid decline in eGFR [11]. The Framingham Heart study comprising 2269 patients and the Third National Health and Nutrition Examination Survey comprising 13,372 subjects showed that the risk of ESRD in participants with serum phosphate levels ≥ 4 mg/dL was approximately 2-fold higher than that in participants with serum phosphate levels < 4 mg/dL [12]. The Ramipril Efficacy In Nephropathy trial that examined the effects of ramipril on 331 patients with CKD showed that serum phosphate levels were an independent risk factor associated with CKD progression against the renotropic effect of angiotensin-converting enzyme inhibitors [13]. Thus, evidence shows that hyperphosphatemia in CKD is associated with poor renal prognoses. Moreover, a study of 803 CKD patients showed that higher serum phosphate levels were associated with an increased risk of ESRD, where the HR was about 17.6 at a time-averaged phosphorus level of 3.4 mg/dL, even in patients with normal serum phosphate levels [14].

Serum phosphate levels are intricately related to *fibroblast growth factor 23* (*FGF23*) and *klotho*, which are key regulators of phosphate homeostasis [15]. *FGF23* reduces the types IIa and IIc sodium-phosphate cotransporters (NaPi-2a and NaPi-2c) in the proximal tubules of the kidneys and the serum 1,25-dihydroxyvitamin D (1,25(OH)_2_D) level, leading to suppressed reabsorption of phosphate [15]. *Klotho* is an important co-factor for *FGF23* and regulates the NaPi-2a and 2c independently of *FGF23* [16].

During early-stage CKD, *FGF23* promotes the excretion of phosphate via the urine while simultaneously suppressing 1,25(OH)_2_D and stimulating the secretion of the parathyroid hormone [17]. Such disorders of mineral metabolism occur in CKD before hyperphosphatemia emerges [18,19]. Elevated *FGF23* and serum phosphate levels have been linked to renal prognoses [17], and a higher *FGF23* level is an independent risk factor associated with ESRD at CKD stages 2 to 3 [18]. In a prospective cohort study of 738 patients with CKD, a combination of low 25-hydroxyvitamin D and high *FGF23* levels predicted lower eGFRs [19]. On the other hand, *klotho* levels gradually decrease according to CKD progression [20].

In one study, *FGF23* levels were found to be 4-fold higher in ADPKD patients at CKD stage 1 to 2 than those in diabetic and non-diabetic CKD patients and healthy volunteers [8]. However, the tubular maximum reabsorption of phosphate (TmP) per unit GFR (TmP/GFR) did not decrease despite excessive *FGF23* secretion in the majority of ADPKD patients, whereas TmP/GFR decreased in X-linked hypophosphatemia patients whose serum *FGF23* levels were lower than those in ADPKD patients [21]. In a previous report, ADPKD patients demonstrated inappropriately high *FGF23* levels compared to the severity of their renal insufficiency [22]. These findings suggested that the biological activity of *FGF23* was reduced in ADPKD patients. In rodent models, resistance to high *FGF23* levels produced in the cyst-lining cells was also reported [23]. Although serum *klotho* levels were low in the majority of ADPKD patients, the levels were significantly lower in the patients whose TmP/GFR did not decrease despite excessive FGF23 secretion [21]. Based on the analyses of *FGF23*-deficient and *klotho*-deficient mice, *klotho* was considered an important factor for accomplishing the biological activity of *FGF23* [24]. Therefore, loss of *klotho* might contribute to *FGF23* resistance in ADPKD patients. Interestingly, serum *klotho* levels were reported to be inversely correlated with cyst volume according to one study [21]. In our study, high serum phosphate levels were significantly associated with poor renal prognoses. This result indicated that loss of *klotho* might affect both high serum phosphate levels and renal prognoses via cyst volume expansion.

The limitations of the study should be considered. Firstly, there was a small sample size, which was a consequence of missing serum phosphate levels for over half of the patients during the initial examination. As a result, a multivariable cox regression model with six renal prognostic associated factors having only 21 patients reaching endpoint was conducted in the present study, which demands careful consideration to interpret the results of the present study. Generally, measurement of serum phosphate levels is recommended for patients with CKD stage 3 and beyond. In our study, among 72 patients who did not undergo serum phosphate measurement during their initial examination, 39 patients (54.2%) were categorized as CKD stages 1 and 2, and 24 patients (33.3%) were categorized as CKD stage 3. In addition, since the study was observational, causality could not be determined. Further studies with a large sample size would contribute to our understanding of these mechanisms and help with clinical interventions to delay the progression of ADPKD.

## 5. Conclusions

This is the first report that found a significant association between serum phosphate levels and poor renal prognosis in patients with ADPKD.

## Figures and Tables

**Table 1:** Patient baseline characteristics according to sex (*n* = 55).

Variables	Entire	Men	Women	*p*–value
	*n* = 55	*n* = 25	*n* = 30	
Clinical Findings				
Age (years, median (Q1, Q3))	49 [40, 59]	49 [41, 54.5]	47.5 [37.5, 64]	0.7543
Sex (Men; %)	25 (45.5)	25 (100)	0 (0)	< 0.0001
SBP (mmHg, median (Q1, Q3))	127.3 [120.5, 135.4]	128.8 [122.1, 136.6]	126 [118.9, 135.1]	0.3252
DBP (mmHg, median (Q1, Q3))	80.3 [74.8, 87.3]	84.7 [75.3, 88.7]	77.2 [71.6, 86.8]	0.1409
MBP (mmHg, median (Q1, Q3))	95.6 [90.3, 102.8]	98.2 [91.6, 103.1]	93.1 [79.3, 102.3]	0.1317
PP (mmHg, median (Q1, Q3))	48 [41.3, 53.2]	50.7 [39.3, 53]	47.3 [43.1, 54]	0.7876
BMI (kg/m^2^, median (Q1, Q3))	22.1 [20.5, 23.4]	23 [21.9, 24.6]	21.1 [19.7, 22.9]	0.0053
Mutation Type				
PKD1 (%)	40 (72.7)	19 (82.6)	21 (72.4)	0.5132
PKD2 (%)	12 (21.8)	4 (17.4)	8 (27.6)	
Laboratory Findings				
Hemoglobin (g/dL, median [Q1, Q3])	12.6 [11.3, 13.6]	13 [11.4, 14.3]	12.5 [11.2, 13.4]	0.1110
Total Protein (g/dL, median [Q1, Q3])	7 [6.8, 7.4]	7.1 [6.8, 7.3]	7 [6.8, 7.4]	0.6633
Serum Albumin (g/dL, median [Q1, Q3])	4.2 [4.1, 4.5]	4.2 [4.1, 4.6]	4.2 [4, 4.4]	0.2262
Blood Urea Nitrogen (mg/dL, median [Q1, Q3])	22.4 [16.5, 32.6]	23.4 [19.1, 40.8]	19.8 [15.8, 29.6]	0.1556
Serum Creatinine (mg/dL, median [Q1, Q3])	1.48 [0.88, 2.7]	1.8 [1.31, 3.99]	1.01 [0.70, 2.34]	0.0035
eGFR (mL/min/1.73m^2^, median [Q1, Q3])	39.5 [17.6, 65.7]	35.6 [14.1, 47.4]	46.6 [18.7, 77.4]	0.0745
Uric Acid (mg/dL, median [Q1, Q3])	6.3 [5.1, 7.2]	7.1 [6, 7.8]	5.4 [4.2, 6.6]	0.0004
Triglyceride (mg/dL, median [Q1, Q3])	109 [74, 167]	137.5 [97.3, 182.5]	79 [59, 135.5]	0.0044
LDL Cholesterol (mg/dL, median [Q1, Q3])	104.6 [82.8, 116.9]	108 [85, 126.8]	100.8 [74.8, 115.9]	0.7042
HDL Cholesterol (mg/dL, median [Q1, Q3])	59 [44, 81.5]	45.2 [36.5, 57]	71 [57.5, 93.5]	0.0049
AST (IU/L, median [Q1, Q3])	18.5 [15.8, 21]	17 [15, 19]	19.5 [16.8, 21.3]	0.0963
ALT (IU/L, median [Q1, Q3])	15 [13, 19.8]	15.5 [12.5, 20.3]	14.5 [12.8, 18.5]	0.6474
ALP (IU/L, median [Q1, Q3])	218 [148, 265.5]	220 [211.5, 294.5]	191 [133.3, 266.8]	0.4555
GGT (IU/L, median [Q1, Q3])	32 [21.5, 52]	36 [26.5, 62]	24 [17.3, 45.3]	0.1602
Sodium (mEq/L, median [Q1, Q3])	141.5 [140, 143]	141 [140, 143]	142 [138.8, 143]	0.5323
Potassium (mEq/L, median [Q1, Q3])	4.3 [4, 4.7]	4.4 [4.1, 4.7]	4.2 [4, 4.8]	0.4111
Phosphorus (mg/dL, median [Q1, Q3])	3.4 [3.1, 3.9]	3.1 [2.8, 4]	3.5 [3.3, 3.9]	0.0903
Calcium (mg/dL, median [Q1, Q3])	9.1 [8.8, 9.3]	9 [8.7, 9.5]	9.2 [8.9, 9.3]	0.5678
Intact PTH (pg/mL, median [Q1, Q3])	195 [140, 244]	171 [135, 244]	211 [143.3, 323.8]	0.7133
Bicarbonate (mEq/L, median [Q1, Q3])	19.6 [18, 23.9]	19.5 [18.3, 24.0]	20.6 [16.5, 24.9]	0.9431
U-Prot (g/gCre, median [Q1, Q3])	0.33 [0.15, 0.57]	0.33 [0.19, 0.56]	0.34 [0.12, 0.63]	0.8601
HbA1c (%, median [Q1, Q3])	5.5 [5.2, 5.9]	5.7 [5.4, 6.0]	5.3 [4.8, 5.8]	0.1244
Concomitant drugs				
Antihypertensive Agents (%)	36 (65.5)	23 (92)	13 (43.3)	0.0002
ARB and or ACEI (%)	26(47.3)	16 (64)	10 (33.3)	0.0223
Calcium Channel Blockade (%)	19 (34.5)	12 (48)	7 (23.3)	0.0872
Others (%)	11 (20)	8 (32)	3 (10)	0.0876
Antihyperuricemic Agents (%)	15 (27.3)	14 (56)	1 (3.3)	<0.0001
Antidyslipidemic Agents (%)	4 (7.3)	3 (12)	1 (3.3)	0.3198
Statin (%)	3 (5.5)	2 (8)	1 (3.3)	0.5855
Others (%)	1 (1.8)	1 (4)	0 (0)	0.4545
Diuretics (%)	7 (12.7)	2 (8)	5 (16.7)	0.4363
Antiplatelets (%)	2(3.6)	0	2 (6.7)	0.4949
EPA or DHA (%)	3 (5.5)	2 (8)	1 (3.3)	0.5855
ESAs (%)	4 (7.3)	2 (8)	2 (6.7)	1.0000
Irons (%)	0 (0.0)	0 (0)	0 (0)	0
Tolvaptan (%)	0 (0.0)	0 (0)	0 (0)	0
Comorbidities				
Hypertension (%)	39 (70.9)	23 (100)	16 (53.3)	< 0.0001
Hyperuricemia (%)	27 (49)	21 (84)	6 (20)	< 0.0001
Dyslipidemia (%)	22 (40)	13 (52)	9 (30)	0.0973
Diabetes (%)	2 (3.6)	2 (8)	0	0.2020

Continuous values were expressed as a median (Q1; Q3). Count data were expressed as n (%). Calcium was expressed as total Calcium concentration (tCa) using Payne’s formula: corrected Ca= tCa + (4-Albumin). Abbreviation: n, number; %, percentages; Q1, quartile 1; Q3, quartile 3; SBP, systolic blood pressure; DBP, diastolic blood pressure; MBP, mean blood pressure; PP, pulse pressure; BMI, body mass index; PKD, polycystic kidney disease; eGFR, estimated glomerular filtration rate; LDL, low-density lipoprotein; HDL, high-density lipoprotein; AST, aspartate aminotransferase; ALT, alanine aminotransferase; ALP, alkaline phosphatase; GGT, gamma-glutamyl transpeptidase; PTH, parathyroid hormone; U-Prot, Urinary protein excretion; HbA1c, Hemoglobin A1c; ARB, angiotensin receptor blocker; ACEI, angiotensin-converting enzyme inhibitor; EPA, eicosapentaenoic acid; DHA, docosahexaenoic acid; ESA, erythropoiesis-stimulating agent.

**Table 2:** Univariate and multivariate Cox analysis of risk factors associated with renal replacement therapy (n = 55).

Variables	Univariate Analysis		Multivariate Analysis	
	Hazard Ratio (95% CI)	*p*–Value	Hazard Ratio (95% CI)	*p-*Value
Age (1 year increase)	1.04 (1.01–1.08)	0.0221	0.96 (0.90-1.02)	0.1790
Men (vs. Women)	1.28 (0.52–3.11)	0.5851	-	-
SBP (10-mmHg increase)	1.12 (0.83–1.48)	0.4565	-	-
DBP (10-mmHg increase)	0.96 (0.61–1.53)	0.8732	-	-
MBP (10-mmHg increase)	1.13 (0.74–1.70)	0.5535	-	-
BMI (1 kg/m^2^ increase)	1.05 (0.88–1.24)	0.5655	-	-
*PKD1* (vs. *PKD2*)	3.61 (1.02–22.96)	0.0468	0.59 (0.07–5.41)	0.6221
eGFR (1-mL/min/1.73m^2^ increase)	0.83 (0.76–0.89)	< 0.0001	0.82 (0.74–0.90)	< 0.0001
Phosphorus (1-mg/dL increase)	4.61 (2.14–10.27)	0.0001	6.78 (1.94–34.02)	0.0021
U-Prot (1-g/g/Cre increase)	1.69 (1.17–2.39)	0.0104	1.92 (0.14–14.90)	0.5869
Hypertension (vs. no)	2.756e^+9^ (5.23-.)	< 0.0001	-	-
Hyperuricemia (vs. no)	3.09 (1.23–8.75)	0.0155	1.11 (0.28–4.89)	0.8812
Dyslipidemia (vs. no)	1.33 (0.55–3.25)	0.5229	-	-
Diabetes (vs. no)	1.462e^-8^(0-.)	0.4043	-	-

Variables with *p*–values of less than 0.1 in the univariate model were included in the multivariate model. Abbreviation: n, number; CI = confidence interval; SBP, systolic blood pressure; DBP, diastolic blood pressure; MBP, mean blood pressure; BMI, body mass index; *PKD*, polycystic kidney disease; eGFR, estimated glomerular filtration rate; U-Prot, urinary protein excretion.
